# Torque Measurement of Coupled Multi-Mechanism Connections for Vertical Replenishment of External Cargo on Shipborne Helicopters

**DOI:** 10.3390/s26165167

**Published:** 2026-08-14

**Authors:** Kai Ma, Haiyang Wang, Menglong Liu, Chung Ming Leung

**Affiliations:** 1 Marine Design and Research Institute of China, Shanghai 200011, China; makai0006102@sina.com (K.M.); dahai1107@163.com (H.W.); 2 School of Robotics and Advanced Manufacturing, Harbin Institute of Technology, Shenzhen 518055, China

**Keywords:** shipborne helicopters, vertical replenishment equipment, torque measurement, wireless sensing, swivel eye

## Abstract

When a shipborne helicopter performs vertical replenishment with external cargo, changes in flight attitude and the combined airflow field may cause the suspended load to rotate, generating torque at the connection assembly. This torque cannot be measured directly during flight, and the interactions among the boom, swivel eye, lifting eye, and cargo frame produce coupled torque components that are difficult to evaluate by simulation alone. Therefore, a real-time torque measurement test system is developed in this study. The system emulates the multi-component connection used in vertical replenishment and can adjust the external load, rotational speed, rotational direction, and offset angle. The tensile force, torque, and rotational speed borne by the swivel eye are measured by a load cell, torque transducer, and tachometer with wireless data transmission. By measuring the torques of a single swivel eye and a double series-connected swivel eye after rotation decoupling, the main torque patterns at the top of the swivel eye are obtained. The results show that the proposed system can measure the torque responses of swivel-eye connections under different loads, offset angles, rotational speeds, and rotation directions. This provides an experimental basis for evaluating the torque transmission behavior of single and double series-connected swivel eyes in shipborne helicopter vertical replenishment.

## 1. Introduction

Vertical replenishment by shipborne helicopters is a core capability for ensuring that naval vessels can carry out long-term missions in the open ocean. Compared to conventional maritime replenishment methods, vertical replenishment offers distinct advantages such as high mobility and transport efficiency. With a maximum single-load capacity of several tons, simple equipment, and minimal manpower requirements, it can operate in rough seas and has become a key technical means for providing mobile replenishment support to aircraft carrier strike groups and amphibious task forces in complex maritime environments. Vertical replenishment missions place extremely high demands on pilots’ precision and the coordination of the flight crew. As maritime operations continue to expand, vertical replenishment technology has been widely adopted for naval replenishment missions and is gradually extending from military applications to civilian sectors such as offshore engineering and emergency rescue.

During helicopter vertical replenishment, the suspended load is subjected to complex and variable torque during hoisting, hovering, transport, and release. These torques arise from coupled factors, including aerodynamic counter-torque generated by the helicopter rotor, operating torque from the main gearbox and tail drive system, load oscillation caused by wind and ship motion, and additional torque in the hook-and-eye system caused by mechanical misalignment. Accurately measuring and evaluating the real-time variation in these torques is important for operational safety, prevention of cargo drop accidents, and avoidance of structural damage to the helicopter. In recent years, studies on torque measurement have mainly focused on three aspects: torque measurement for multi-coupled connection devices, torque measurement for heavy loads with angular misalignment, and torque measurement for rotating equipment in flight [1,2,3].

Torque measurement for multi-coupled systems is primarily aimed at meeting the torque detection requirements of helicopter vertical replenishment hooks and external suspension equipment (including suspension booms, swivel eyes, lifting eyes, and cargo frames) under complex force coupling conditions. Helicopter vertical replenishment operations involve multiple influencing factors, such as multi-axis coupling effects, dynamic responses of suspended loads, and environmental disturbances, which pose significant challenges to accurate torque decoupling and measurement. This research focuses on torque measurement for components such as hooks, boom arms, swivel eyes, lifting eyes, and cargo frames under complex force coupling conditions. Accurate decoupling and measurement are challenging due to multi-axis coupling, dynamic load responses, and environmental disturbances. Six-axis force/torque sensors are a key approach [4,5], with measurement methods including strain gauges, piezoelectric, capacitive, and photoelectric sensors. Cao et al. reviewed the design principles and calibration methods of six-axis force/torque sensors for robotics applications [6]. By the arrangement of T-shaped structures symmetrically around the upper and lower parts, and the development of electrical and electronic circuits based on cheap microcontrollers, Ha et al. designed a wireless, user-friendly six-axis sensor that overcomes the reliability and high-cost issues of traditional sensors [7]. Wolstrup et al. reviewed performance optimization strategies for 3D-printed electromechanical sensors, and recommended focusing on aligning sensing mechanisms with appropriate fabrication strategies and aiding metric standardization across the field [8]. Jiang et al. developed a torsion spring-type torque sensor based on a dual rotary transformer, and solved the problems of unequal amplitude and nonorthogonal phase of the excitation signal [9]. Stiglmeier et al. reviewed 3D-printed torque sensors, noting that additive manufacturing can reduce costs and complexity [10]. For wireless measurement, wireless strain gauge telemetry systems offer significant advantages in rotating shaft power testing [11]. Ren et al. achieved non-contact torque measurement of a rotating flywheel using wireless telemetry resistive strain gauges [12]. A wireless instantaneous torque system based on MEMS accelerometers and high-frequency RF provides a low-power solution for the simultaneous measurement of torque and rotational speed on rotating shafts [13]. Misalignment faults in rotating machinery can also be identified using torque signals, demonstrating that torque measurement can complement traditional vibration measurement [14], which offers valuable insights for the online monitoring of vertical replenishment systems.

The measurement of torque under large off-center loads is primarily aimed at addressing the issue of additional torque generated by load eccentricity and misalignment of external lifting-eye equipment during vertical replenishment operations. In offshore operations, where loads can range from hundreds to thousands of kilograms, a horizontal offset exists between the hook and the center of gravity; the resulting off-center torque is transmitted to the hook, the main gearbox, and the airframe, imposing an additional burden. Regarding dynamic modeling, Sansal et al. investigated the impact of suspended load coupling dynamics on helicopter control quality [15]. Tian et al. proposed a nonlinear rigid-elastic coupling modeling method to uniformly describe rotor–airframe–suspension coupling oscillations [16]. Oktay et al. proposed a fuzzy gain scheduling adaptive anti-sway strategy to balance load sway suppression and command tracking [17]. Wang et al. investigated adaptive disturbance-rejection control with friction compensation, providing a compensation framework for torque control under offset-angle conditions [18]. Lu et al. used simulations to clarify the effects of duct presence or absence on shaft rotational torque [19]. In terms of sensing and detection, a torque calibration system based on phase-shift measurement calculates torque by monitoring shaft angular deflection, demonstrating good adaptability to offset-angle conditions [20]. Li et al. conducted research on dynamic modeling and vibration control for cooperative lifting by twin helicopters [21]. Under heavy loads, load sway and yaw, transmitted to the airframe via the eyes, can cause dynamic imbalances in main rotor torque and tail rotor counter-torque. The feedforward-feedback combined control strategy developed by Bisgaard et al., with its full-state estimator, provides a theoretical foundation for yaw angle torque compensation [22].

Torque measurement for rotating aerial equipment primarily focuses on dynamic torque detection in high-speed rotating helicopter rotor systems (main rotor and tail rotor), as well as torque monitoring during power transmission between rotating components. The technical challenge in this area lies in the need for sensors to achieve precise, non-contact, real-time measurements under harsh conditions characterized by high rotational speeds, strong vibration, and limited installation space [23,24]. Regarding airframe-level measurements, Bossler reviewed four primary methods—hydraulic pressure, gear eye phase displacement, changes in material properties, and telemetric strain gauges [25]. In the field of magneto elastic sensing, high-bandwidth sensors from Methode Inc. allow helicopters to operate near their transmission limits [26]; non-contact sensors from MagCanica Inc. provide laboratory-grade accuracy at speeds as high as 140,000 rpm [27]. Non-contact torque sensors for tail rotor drive systems have also been developed recently with two magnet rings as the sensing coils on the shaft [28]. Regarding wireless passive sensing, Li et al. proposed battery-free wireless sensors powered by RF energy to enable long-term real-time monitoring [29]. Regarding tail drive monitoring, the Bell 525 helicopter uses tail rotor torque measurement data to support maintenance certification, and flight tests have verified its high accuracy [30]. Engineering practice shows that the lack of torque monitoring can cause helicopters to be constrained by conservative power limits, whereas high-bandwidth sensors allow them to operate safely near the limits of the transmission system [31].

Although significant progress has been made in the aforementioned studies [32], the torque behavior of shipborne helicopter vertical replenishment connections has not been systematically characterized from the perspective of real-time sensing. In this scenario, the measured torque has three notable characteristics. First, the ship wake and rotor downwash produce unsteady airflow disturbances during hover that are stronger than those in conventional flight. Second, the changing offset angle between the hook point and the load center of gravity generates additional moments and changes the torque transmitted through the connector. Third, the elastic response of the lifting eye and the sagging of the suspended load may lead to transient torque fluctuations, requiring sufficient sensor response and acquisition accuracy. Therefore, a method for measuring the torque at the connection point between the external load-carrying equipment and the helicopter is needed to support the torque design of the equipment and its connection points.

Addressing the above issues regarding the rotation cancelation, two kinds of swivel eyes have been designed, i.e., a single swivel eye and double series-connected swivel eye, and a real-time wireless torque measurement system is proposed to measure the value of torque in shipborne helicopter vertical replenishment. In this system, tensile loading, offset-angle adjustment, rotational control, and wireless torque sensing are combined in one test platform. The torque responses of single and double series-connected swivel eyes were then measured under different loading cases. By comparing these results, the torque transmission behavior of the swivel-eye connections can be evaluated, providing an experimental basis for the design and safety assessment of vertical replenishment connection assemblies, to make sure that the maximum torque under the severe working conditions is lower than the threshold.

The rest of the paper is organized as follows. Section 2 introduces the principle of rotation cancelation in vertical replenishment, followed by the design of the torque measurement system in Section 3. Test results and discussion are presented in Section 4, and concluding remarks are summarized in Section 5.

## 2. Principle of Rotation Cancelation in Vertical Replenishment

**Figure 1:**
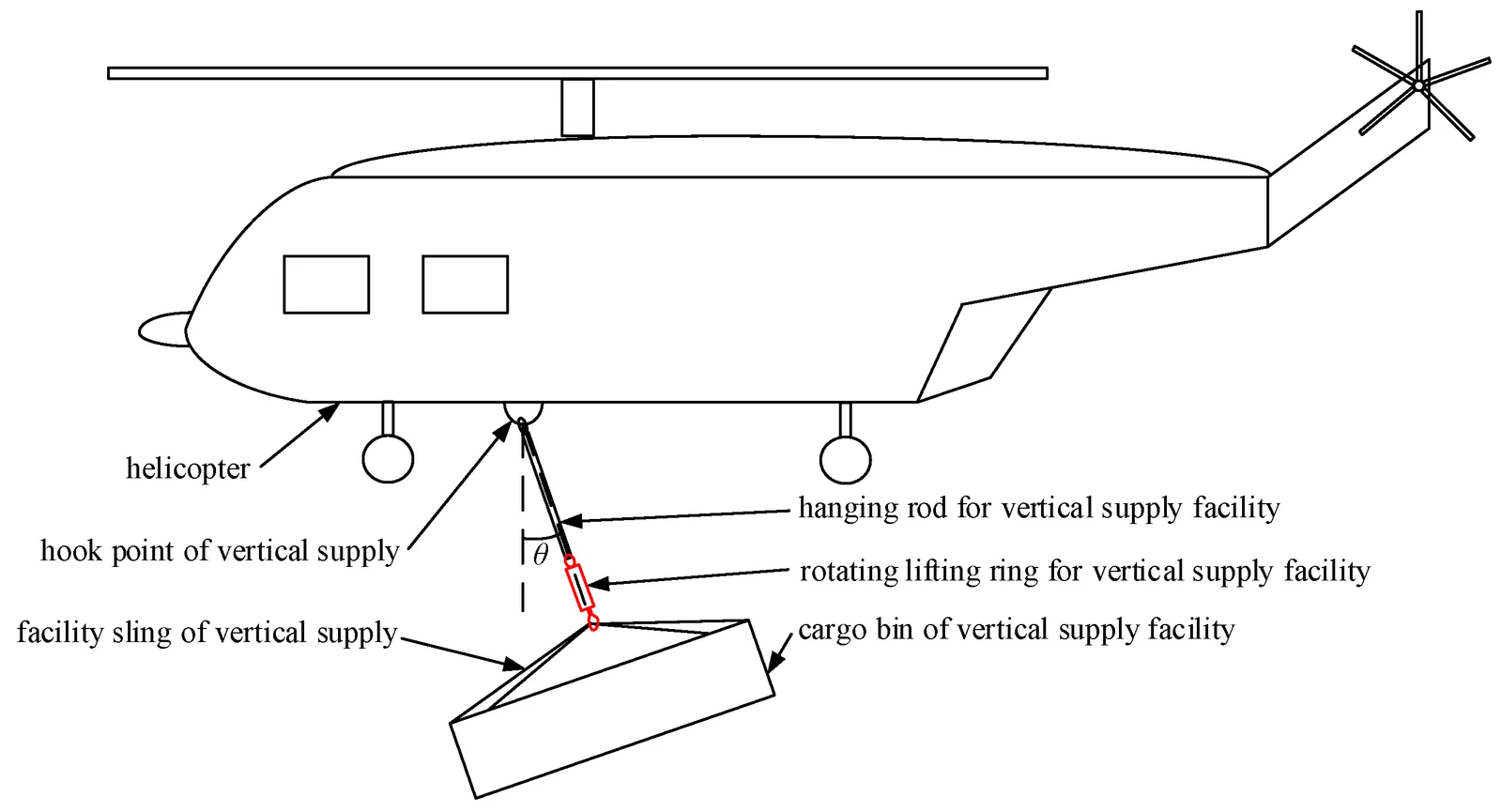
Schematic diagram of vertical replenishment for a shipborne helicopter.

Vertical replenishment for shipborne helicopters is usually carried out using a vertical replenishment system that connects the helicopter hook to the cargo container, as shown in Figure 1. The bottom of the swivel eye directly bears the torque generated by the combined effects of helicopter flight, aerodynamic disturbance, and cargo rotation. After part of the torque is reduced by the anti-rotation mechanism, the remaining torque is transmitted to the helicopter. Measuring the torque at the top of the swivel eye can therefore support the design of the connection between the helicopter and the vertical replenishment system. For the swivel eye used in this test, the performance specification is that, when a shipborne helicopter carries out vertical replenishment with a maximum suspended load of 3 tons and the load rotates at a speed up to 1 rev/4 s, the torque transmitted to the top of the swivel eye shall not exceed 100 N·m.

**Figure 2:**
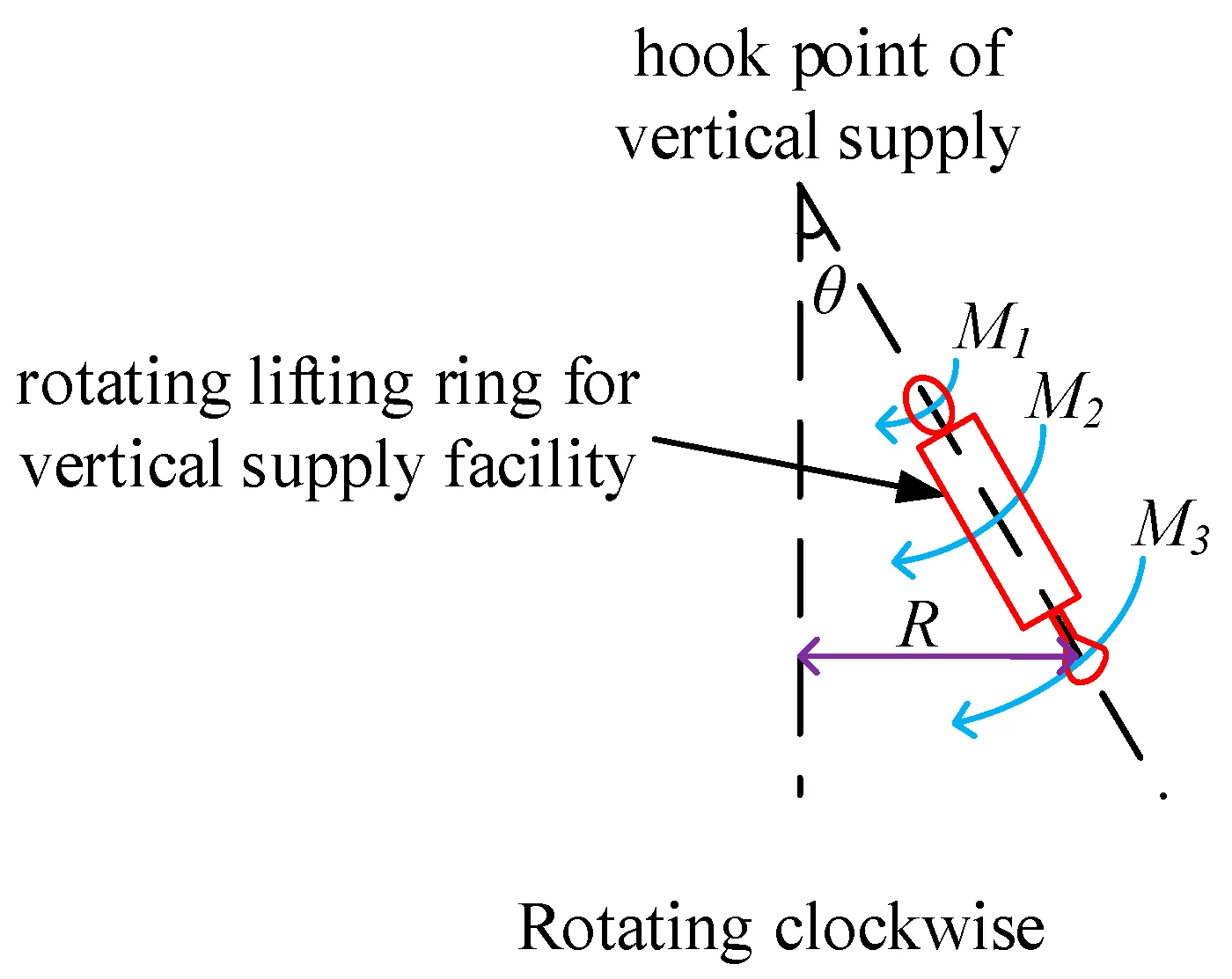
Schematic diagram of torque relationships.

As shown in Figure 2, the torque relationship for the vertical replenishment equipment is given by the following formula:

(1) $$M_{1}=M_{3}-M_{2}$$

where *M*_1_ represents the torque at the top of the swivel eye; *M*_2_ is the torque counteracted by the swivel eye; and *M*_3_ represents the torque at the bottom of the swivel eye.

**Figure 3:**
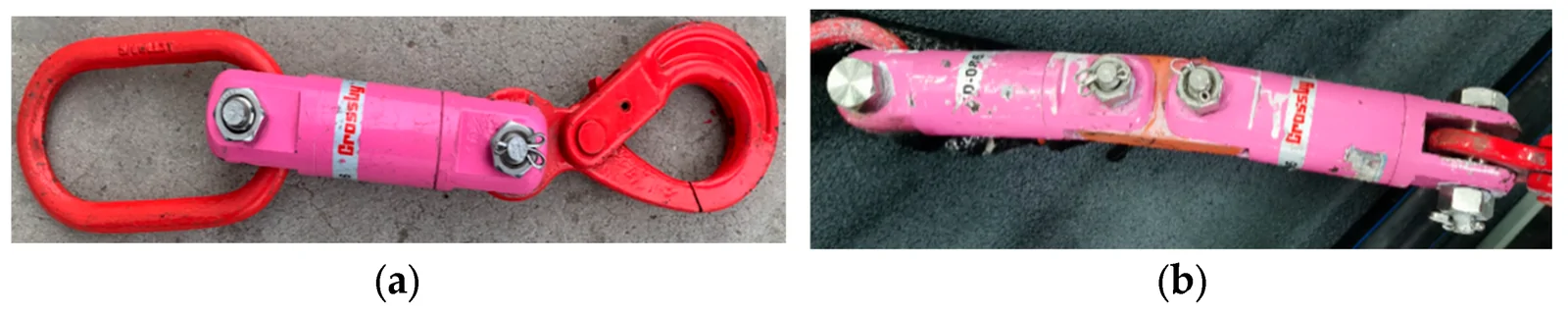
Photographs of swivel eyes: (**a**) a single swivel eye and (**b**) a double series-connected swivel eye.

The photographs of a single swivel eye and a double series-connected swivel eye are shown in Figure 3. The single swivel eye shown in Figure 3a consists of an oval eye at the top, an anti-rotation mechanism in the middle, and a hook at the bottom, all connected by bolts. The double series-connected swivel eye shown in Figure 3b consists of an oval eye at the top, two anti-rotation mechanisms in the middle, and a hook at the bottom, all connected by bolts.

## 3. Torque Measurement System

### 3.1. Apparatus of the Torque Measurement System

**Figure 4:**
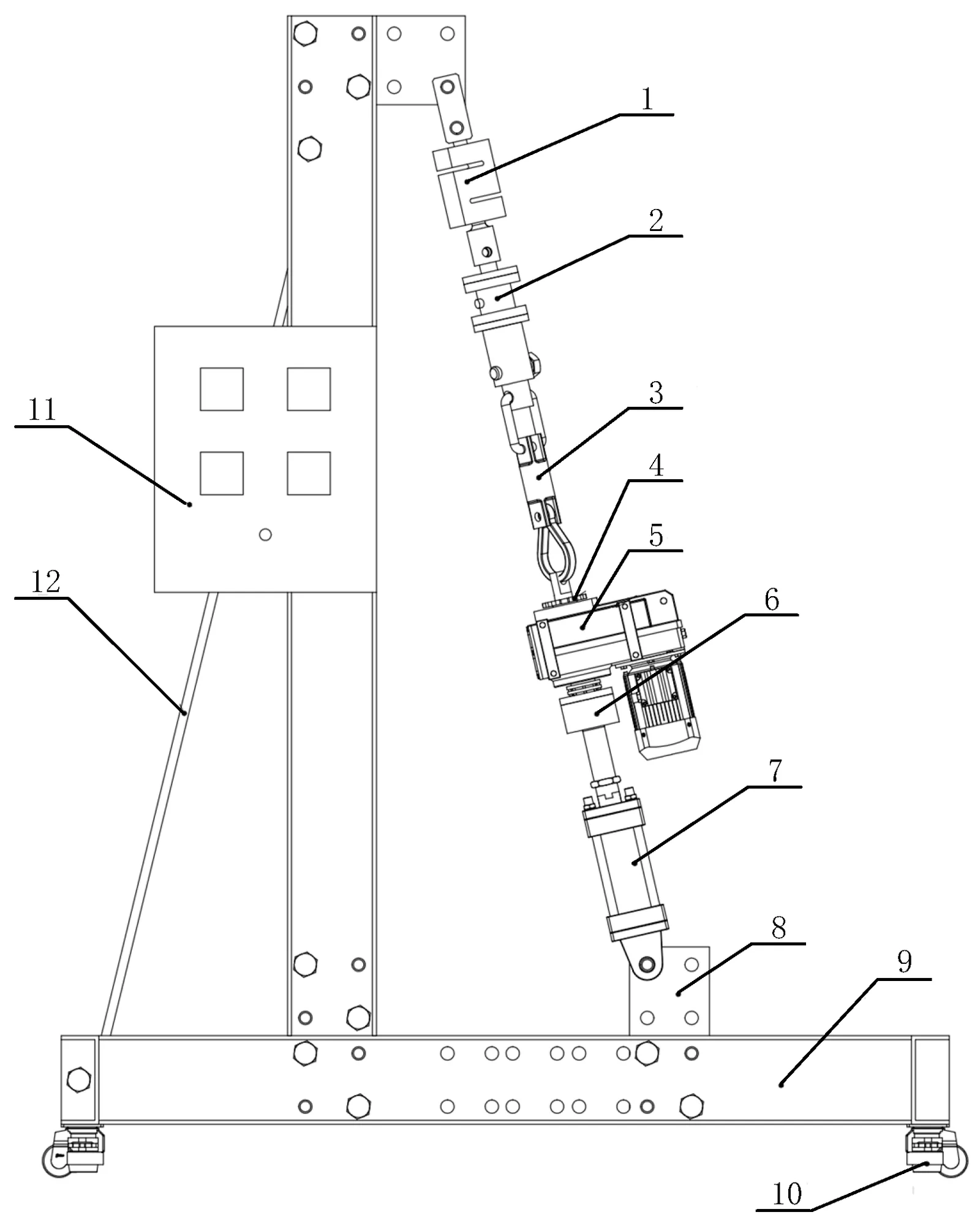
Schematic diagram of a real-time torque measurement test system: 1 force transducer, 2 torque transducer, 3 rotating test eye, 4 tachometer, 5 speed-regulating motor, 6 rotating mechanism, 7 hydraulic cylinder, 8 offset angle adjustment plate, 9 tensioning frame, 10 pulley, 11 test data processing system including DH5908L dynamic strain gauge and data processing computer, and 12 tensile test wire rope.

A real-time torque measurement test system was designed, as shown in Figure 4. The loading system (GGJ04, Zhongjike Vehicle Testing Engineering Research Co., Beijing, China) uses an angle adjustment plate assisted by a tensioning frame, a hydraulic cylinder and a variable-speed motor. The rotational speed and direction are controlled by a variable-frequency drive, allowing different offset angles, tensile forces, and rotational speeds to be set during rotation of the test fixture. The key components of the torque measurement system are as follows: (1) a manually operated digital inclinometer with display; (2) a force transducer with wireless transmission; (3) a tachometer that controls the rotational speed from 0 to 50 rpm, with a measurement error of less than 1%, wireless transmission, and display; (4) a static double-flange torque sensor, with wireless transmission; (5) a display meter, showing the offset angle, tensile force, rotational speed, and torque at top of the swivel eye; and (6) the dynamic measurement system integrating the dynamic strain gauge which receives the voltage signal through Wi-Fi and converts to measured tensile force and torque, and a computer to store and display all the data.

In addition to the devices used to simulate offset angle, apply tensile force, control rotational speed, and measure the key parameters, the auxiliary equipment in the torque measurement test system has the following functions. (1) The rotary mechanism connects the speed-regulating motor to the hydraulic cylinder and counteracts the rotational effect of the motor on the hydraulic cylinder. (2) The tensioning frame assists in adjusting the rotational offset angle and supports the weight of the test system and working load. (3) The roller is used for horizontal movement and braking of the test system. (4) The tension wire rope is used to reduce the influence of simulated load rotation on the torque measurement test system.

This test system has the following features. For communication, a standard 2.4 GHz wireless Wi-Fi technology with a line-of-sight range of up to 200 m is adopted to enable remote and synchronized measurement, reduce on-site workload, and improve testing safety. An optional GPS/Beidou navigation module can be used for synchronized sampling across all modules. Wireless communication also allows the system to be installed close to the measurement point, shortening the signal cables and reducing line interference and installation workload. In addition, the system includes built-in DC bridge supply and signal acquisition/processing circuits, and the digital signals are transmitted wirelessly to reduce external interference. The system can also be directly controlled by a laptop connected to the software, allowing real-time data display.

The main parameters of the dynamic strain gauge (DH5908L, Donghua, Changzhou, China) are as follows. To support operation in harsh environments such as rain, the system reaches an IP65 protection rating. Each computer can simultaneously control 16 modules, and the sampling rate can reach 50 kHz. The operating voltage range can be up to ±5 V, and the corresponding strain ranges include ±30,000 με. The torque sensors include built-in standard resistors, and the software-programmable bridge configurations include full-bridge and half-bridge modes with freely adjustable resistors from 60 Ω to 20,000 Ω, as well as 3-wire 1/4-bridge configurations of 120 Ω or 350 Ω. In addition, the system has a bridge self-test function and allows any measurement point to be designated as a compensation point. Real-time data acquisition, display, storage, and analysis are supported, and a built-in high-capacity lithium battery provides continuous operation for more than 12 h.

The measurement ranges and parameters of this test system are listed in Table 1 and Table 2. With a wide range of operating conditions, the system meets the requirements for different test scenarios involving rotation of the swivel eye.

### 3.2. Principle of Torque Measurement for a Rotating Swivel Eye

**Figure 5:**
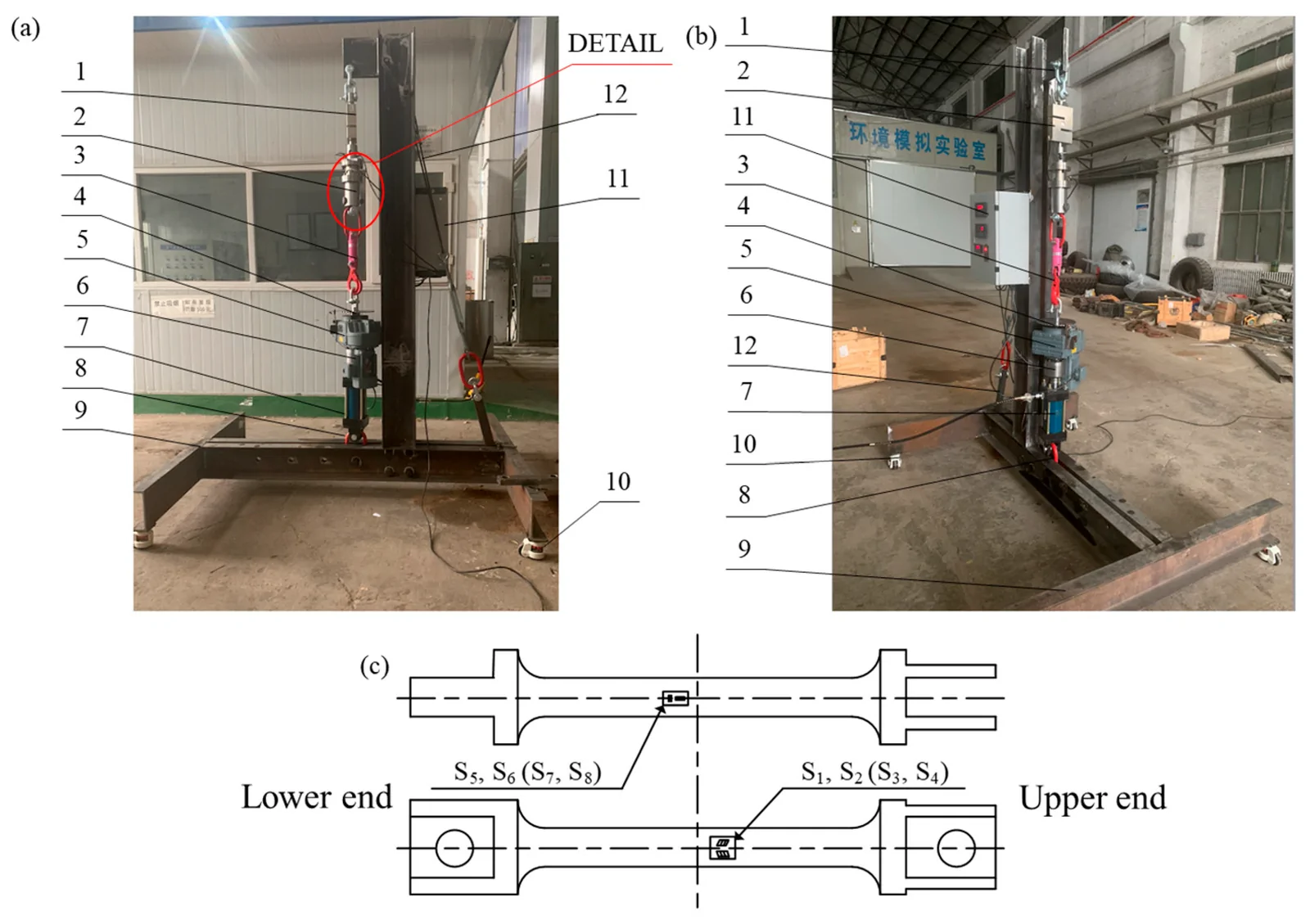
Experimental measurement of torque: (**a**) single swivel eye, (**b**) double series-connected swivel eye, and (**c**) DETAIL: Schematic diagram of strain measurement point layout for the sensitive element of a torque sensor: 1 force transducer, 2 torque transducer, 3 rotating test eye, 4 tachometer, 5 speed-regulating motor, 6 rotating mechanism, 7 hydraulic cylinder, 8 offset angle adjustment plate, 9 tensioning frame, 10 pulley, 11 test data processing system, and 12 tensile test wire rope.

The experimental measurement setup is shown in Figure 5a,b. Considering the load characteristics and technical specifications of the swivel eye, a specialized torque sensor composed of multiple strain gauges is designed to measure torque and tensile force, as illustrated in Figure 5c. The lower end of the torque sensor has a single-ear design and is connected to the upper double-ear interface of the swivel eye through a pin. The upper end has a double-ear configuration and is connected to the lifting eye through a pin. An aluminum foil resistive strain gauge (ZF350-2HA, Zhonghang Electronic Measuring Instruments Co., Ltd., Xi’an, China) is mounted on a cross-section at the center and arranged at 180° intervals. It consists of two sets of ±45° strain gauges numbered S1–S4, which are oriented at ±45° relative to the axis to measure the shear strain generated by torque and form a full-bridge configuration for torque measurement. At another cross-section in the middle, an orthogonal bidirectional aluminum foil resistive strain rosette (BF350-3BB, Zhonghang Electronic Measuring Instruments Co., Ltd., Xi’an, China) is arranged at 180° intervals and numbered S5–S8. It consists of two 0° and two 90° strain gauges, which are used to measure axial tensile load and form a full bridge for tensile-load measurement.

**Figure 6:**
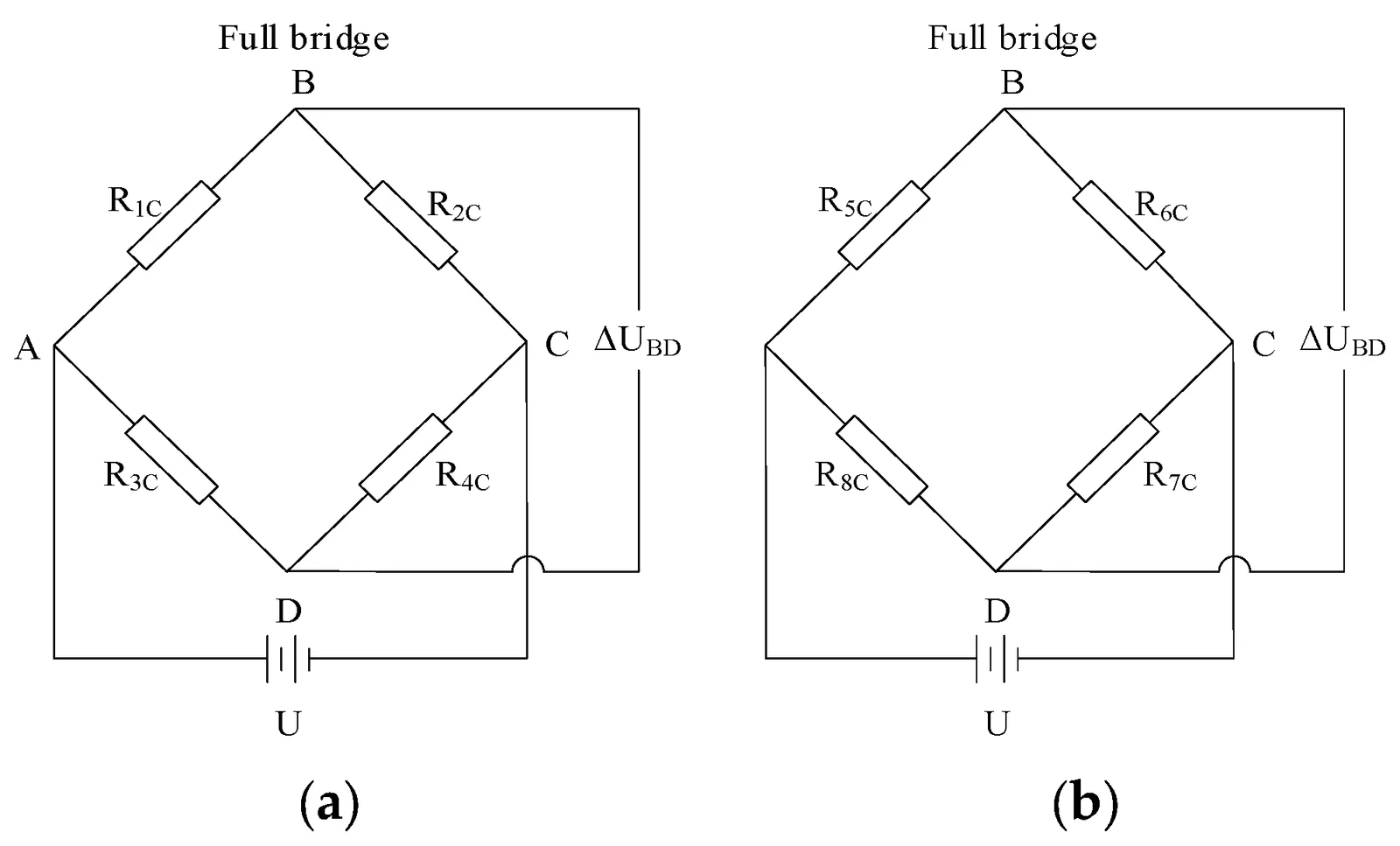
Schematic diagram of strain gauge bridge configuration: (**a**) full bridge to measure the strain due to torque and (**b**) full bridge to measure the strain due to tensile force.

The strain measurement points are configured as a Wheatstone bridge, whose bridge configuration is shown in Figure 6. The full-bridge strain-resistance measurement circuit used in the torque measurement test system is based on the strain-resistance effect, which converts mechanical deformation into changes in electrical signals. When the torque sensor is subjected to force and deformation, the attached strain gauges are stretched or compressed, and the slight change in the geometric dimensions of the metal foil causes a change in resistance. Four strain gauges with identical resistance-change rates form the four arms of a Wheatstone bridge. Initially, the resistance values are balanced, and no output is generated. When force is applied, the resulting deformation disrupts this balance: gauges under tension experience increased resistance, while gauges under compression experience decreased resistance. This produces a differential voltage that is linearly proportional to strain. The differential voltage can be converted into the torque exerted on the upper end of the swivel eye. This full-bridge strain-resistance circuit provides high sensitivity, temperature compensation, measurement accuracy, and interference suppression, meeting the requirements for torque measurement under heavy loads with different offset angles and rotational speeds.

The coefficient between the torque and the output voltage is derived as follows. When a tensile force *F* and torque *T* are applied to the shaft of the sensor, the normal and shear stresses on the outer surface are

(2) $$\left\{\begin{array}{c} \sigma =\frac{F}{A}\\ \tau =\frac{T\cdot r}{J} \end{array}\right.$$

where *A* denotes the cross-sectional area, *r* denotes the radius, and *J* denotes the polar moment of inertia. Since the gauges are bonded on the outer surface of the sensor shaft, the outer-surface radius is used for the strain calculation, and substitution gives

(3) $$\tau =\frac{T\cdot \left(D/2\right)}{\frac{\pi }{32}\left(D^{4}-d^{4}\right)}=\frac{16TD}{\pi \left(D^{4}-d^{4}\right)}$$

where *D* and *d* are the outer and inner diameters, respectively. In a state of combined shear and tensile loading, the normal stresses at ±45° to the shaft axis can be expressed as

(4) $$\sigma_{\pm 45^{0}}=\pm \tau +\frac{\sigma }{2}$$

Using Hooke’s law for plane stress, the normal strains along the ±45° direction are

(5) $$\left\{\begin{array}{c} \varepsilon_{+45}=\frac{\tau \left(1+v\right)}{E}+\frac{\sigma \left(1-v\right)}{E}=\varepsilon_{\tau }+\varepsilon_{\sigma }\\ \varepsilon_{-45}=\frac{-\tau \left(1+v\right)}{E}+\frac{\sigma \left(1-v\right)}{E}=-\varepsilon_{\tau }+\varepsilon_{\sigma } \end{array}\right.$$

For a full bridge, the differential output voltage is generally given by

(6) $$V_{0}=\frac{V_{s}}{4}\cdot GF\cdot \left(\varepsilon_{1}-\varepsilon_{2}+\varepsilon_{3}-\varepsilon_{4}\right)$$

Substituting the above strain expressions into the full-bridge output-voltage equation gives

(7) $$V_{0}=V_{s}\cdot GF\cdot \varepsilon_{\tau }$$

Hence, the voltage output is independent of the tensile loading. Finally, the torque sensitivity coefficient *K* can be calculated as

(8) $$K=\frac{V_{0}}{T}=\frac{16V_{s}\cdot GF\cdot D\left(1+\nu \right)}{\pi E\left(D^{4}-d^{4}\right)}$$

**Figure 7:**
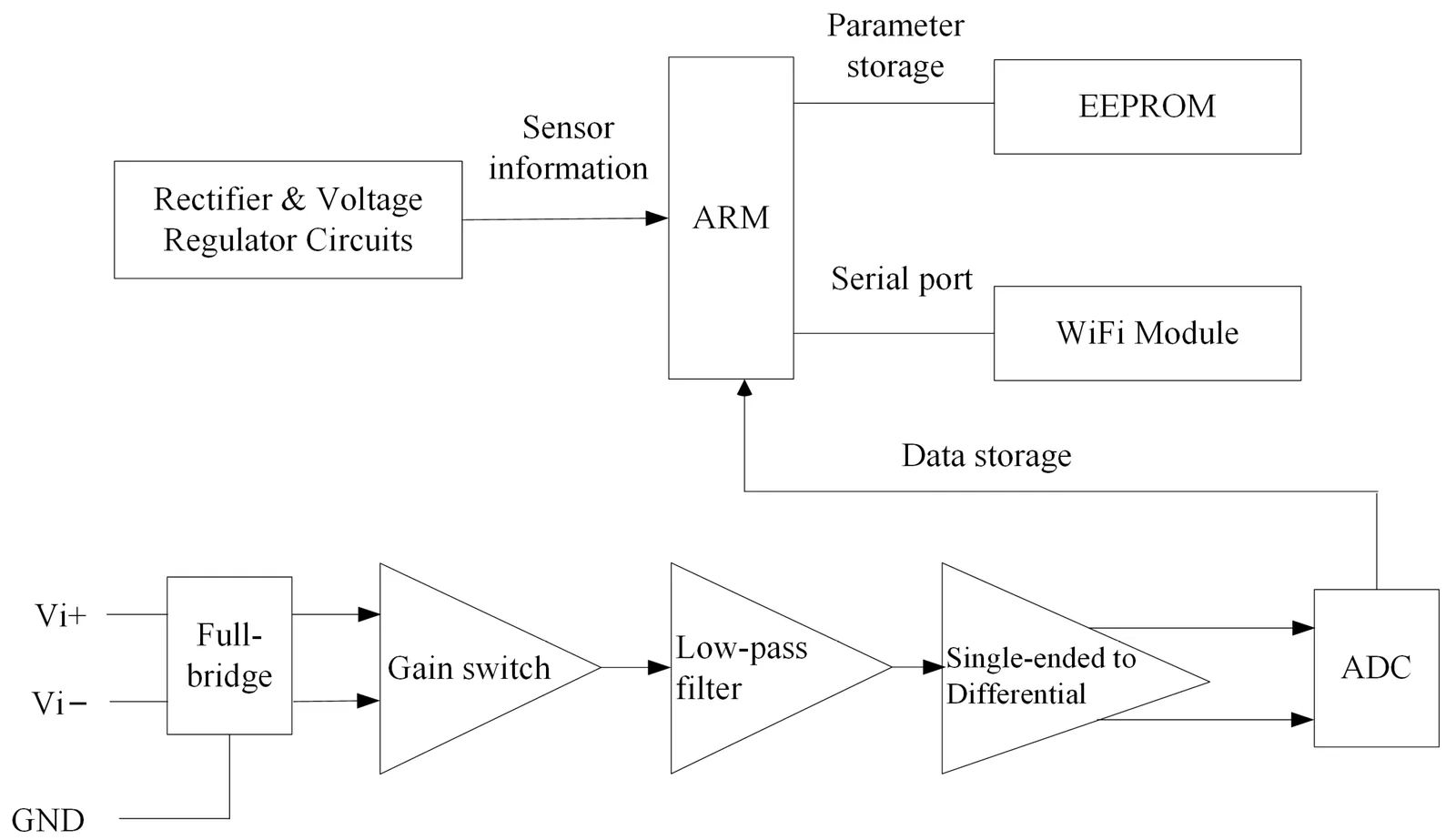
Illustration of electrical circuit for the measurement of strain signal.

Because a commercial loading system (GGJ04, Zhongjike Vehicle Testing Engineering Research Co., Beijing, China) is used, the coefficient *K* was provided by the manufacturer and used as the reference coefficient in this study. To acquire strain signals, the DH5908L wireless dynamic strain gauge is used to capture strain data from the torque sensor. The electrical circuit for strain-signal measurement and storage and the technical specifications are shown in Figure 7 and listed in Table 3, respectively.

### 3.3. Test Setup for Torque Measurement

**Figure 8:**
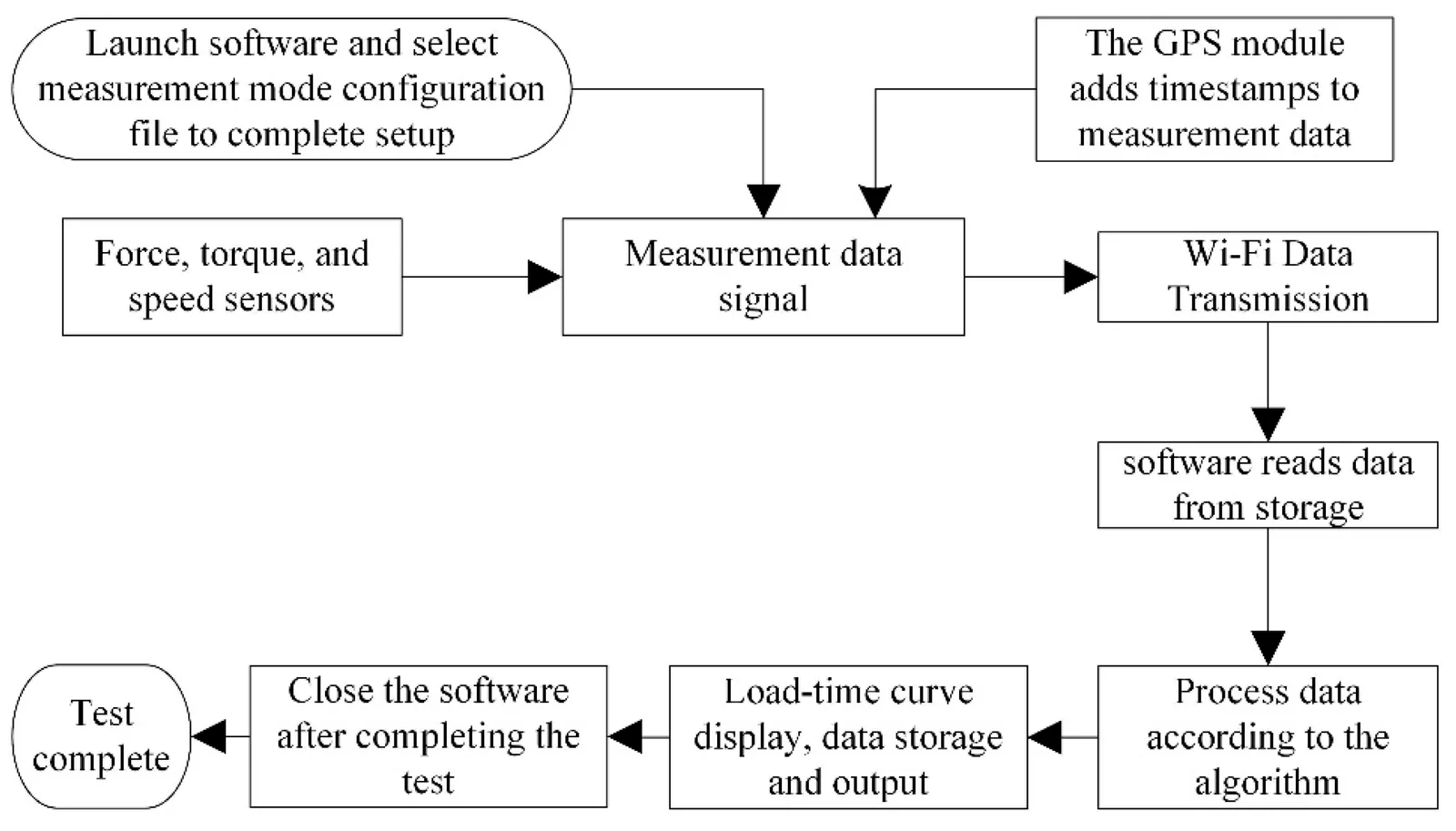
Flowchart of torque measurement.

The flowchart of torque measurement is shown in Figure 8. Based on the design of the torque measurement test system described above, the system is manufactured and assembled. The torque transducer with bonded strain gauges is mounted at the top of the swivel eye. The DH5908L dynamic strain gauge and the central processing computer are turned on, and the DH5908L measurement software is launched on the central processing computer with the measurement-mode configuration file loaded. The measured tensile force, rotational speed, and torque are transmitted to the DH5908L dynamic strain gauge, and the measurement data are time-stamped through the GPS/Beidou module. The data are transmitted wirelessly via Wi-Fi to the central processing computer. The DH5908L measurement software reads the data, processes them using the preset algorithms, and displays and outputs the load-time curve. After measurements under different operating conditions are completed, the measurement equipment, sensors, software, and central processing computer are shut down.

After the test system is assembled and verified, torque measurements are conducted at the top of the single swivel eye and the double series-connected swivel eye. The torque generated during vertical loading is measured for both single-swivel-eye and double series-connected swivel-eye configurations, as shown in Figure 5.

Taking the torque measurement of a single swivel eye under a 2-ton load as an example, the following steps are followed:

(1) Assemble the swivel eye with a 0° offset angle using the angle adjustment plate and tension frame;
(2) Use a hydraulic cylinder to apply a 2-ton tensile force to simulate the weight of a vertical external suspension device;
(3) Set the variable-speed motor to rotate clockwise at 1 rev/16 s, 1 rev/12 s, 1 rev/8 s, and 1 rev/4 s to measure the torque borne by the top of the swivel eye;
(4) Set the variable-speed motor to rotate counterclockwise at 1 rev/16 s, 1 rev/12 s, 1 rev/8 s, and 1 rev/4 s to measure the torque borne by the top of the swivel eye;
(5) Using the angle adjustment plate and tension frame, set the offset angles to 5°, 10°, and 15°, respectively, and then assemble the individual swivel eye;
(6) Repeat steps 3 to 5 to measure the torque borne by the top of the swivel eye;
(7) Repeat steps 1 to 6 for two additional repeated tests.

After the 2-ton load test, the load is increased to 3 tons, and the above steps are repeated. This allows the torque of a single swivel eye under a 3-ton load to be measured at offset angles of 0°, 5°, 10°, and 15°, and at clockwise and counterclockwise rotational speeds of 1 rev/16 s, 1 rev/12 s, 1 rev/8 s, and 1 rev/4 s.

For the double series-connected swivel eye, the torque measurements under loads of 2 tons and 3 tons are conducted using the method applied to the single swivel eye. The tests are performed at an offset angle of 15° and at rotational speeds of 1 rev/16 s, 1 rev/12 s, 1 rev/8 s, and 1 rev/4 s in both clockwise and counterclockwise directions.

## 4. Results and Discussion

### 4.1. Torque of a Single Swivel Eye

**Figure 9:**
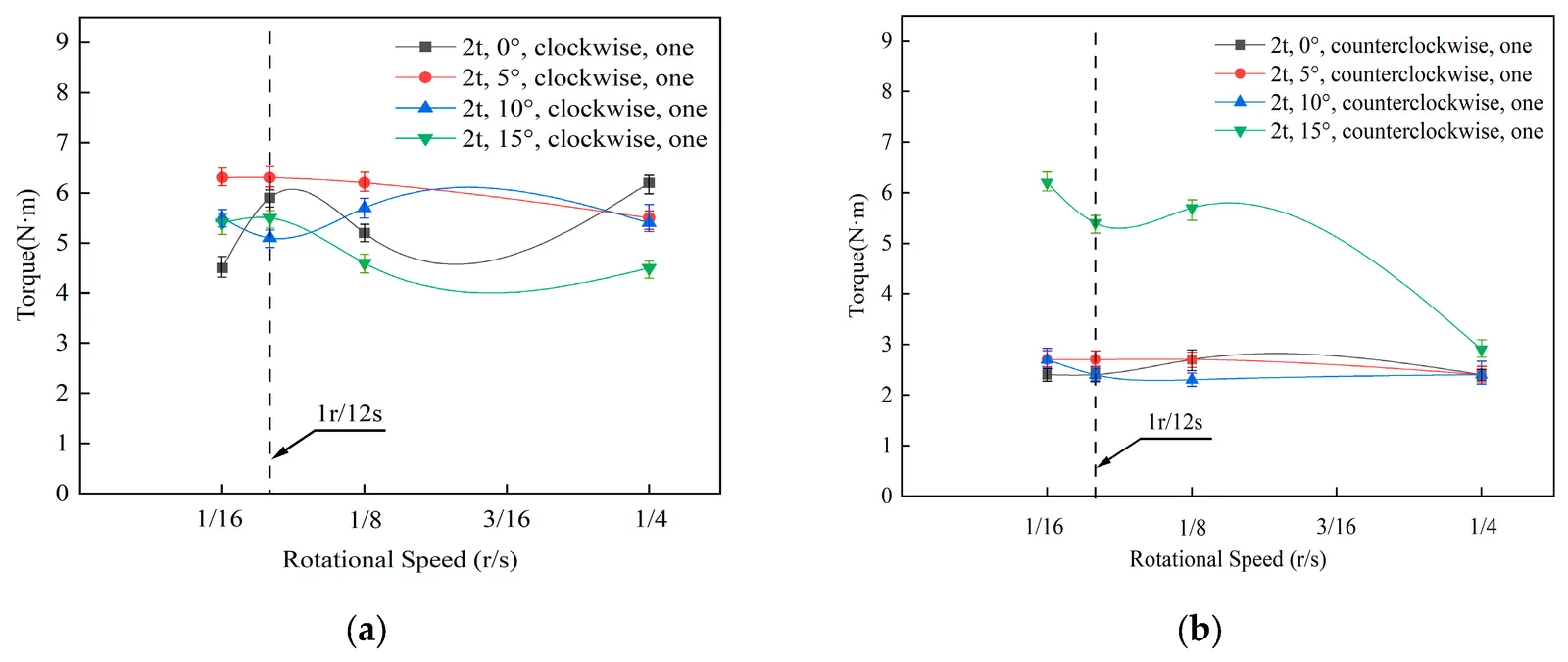
Torque variations in the single swivel eye under the 2-ton load: (**a**) clockwise rotation and (**b**) counterclockwise rotation.

**Figure 10:**
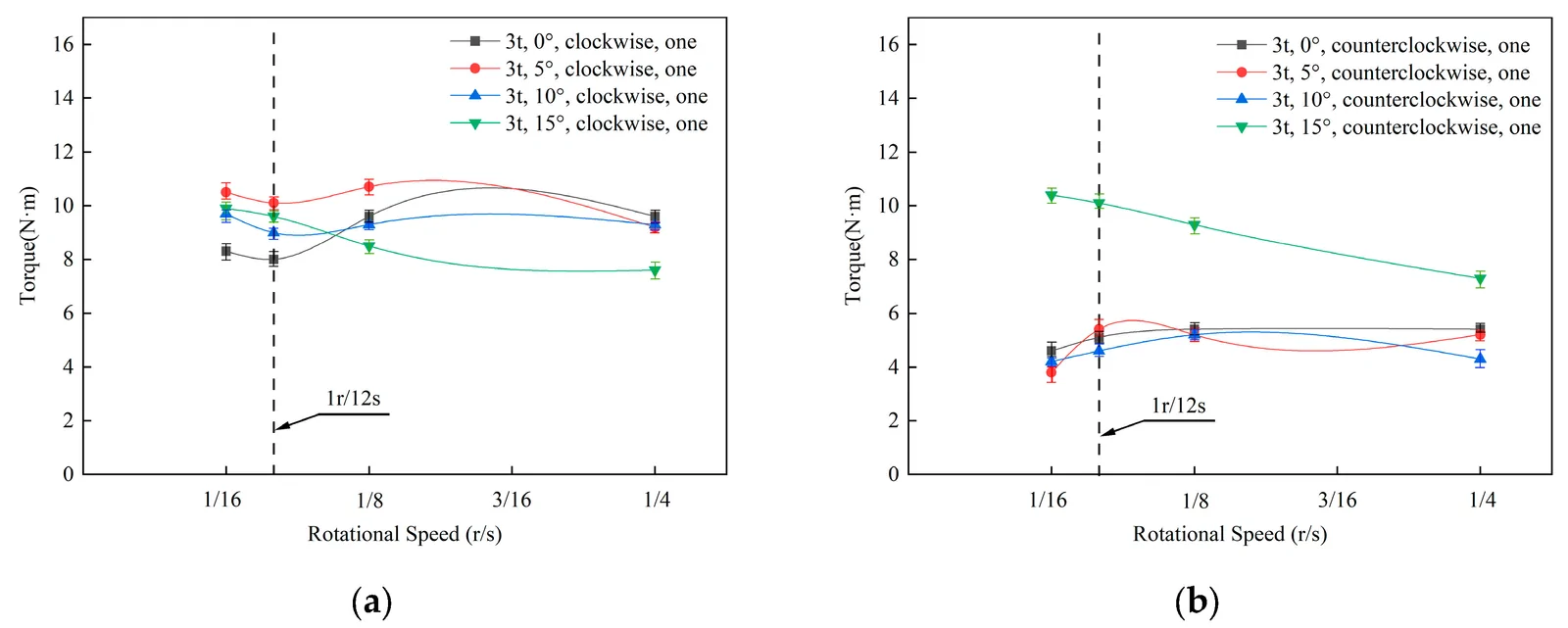
Torque variations in the single swivel eye under the 3-ton load: (**a**) clockwise rotation and (**b**) counterclockwise rotation.

Measurement results for a single swivel eye are evaluated under the 64 conditions described in Section 3.3, with the results shown in Figure 9 and Figure 10, and listed in Table 4 and Table 5. Note that only the magnitudes of the measured torques are concerned; all the torques are presented with their absolute values.

As shown in Figure 9 under the 2-ton load, during clockwise rotation, the rotational speed and offset angle have little influence on the torque. However, during counterclockwise rotation, the torque at the 15° offset angle is greater than that at the 0°, 5°, and 10° offset angles. Under the same offset angle no greater than 10°, clockwise rotation generally produces greater torque than counterclockwise rotation.

As shown in Figure 10, the torque trends for the 33-tonload during clockwise and counterclockwise rotation at the same offset angle are similar to those for the 2-ton load, but the measured values are higher. The torque range for the 3-tons load is between 3 and 11 N·m. Under the same offset angle no greater than 10°, clockwise rotation generally produces greater torque than counterclockwise rotation.

In summary, when a single swivel eye rotates, the torque increases with the load under the same rotational direction and offset angle, while the overall trend remains similar. Under the same load and offset angle no greater than 10°, the torque in the clockwise direction is generally greater than that in the counterclockwise direction, with values ranging from 2 to 11 N·m. Under all the loading conditions, the maximum torque transmitted to the top of the single swivel eye is far below the specified limit of 100 N·m.

### 4.2. Torque of Double Series-Connected Swivel Eye

**Figure 11:**
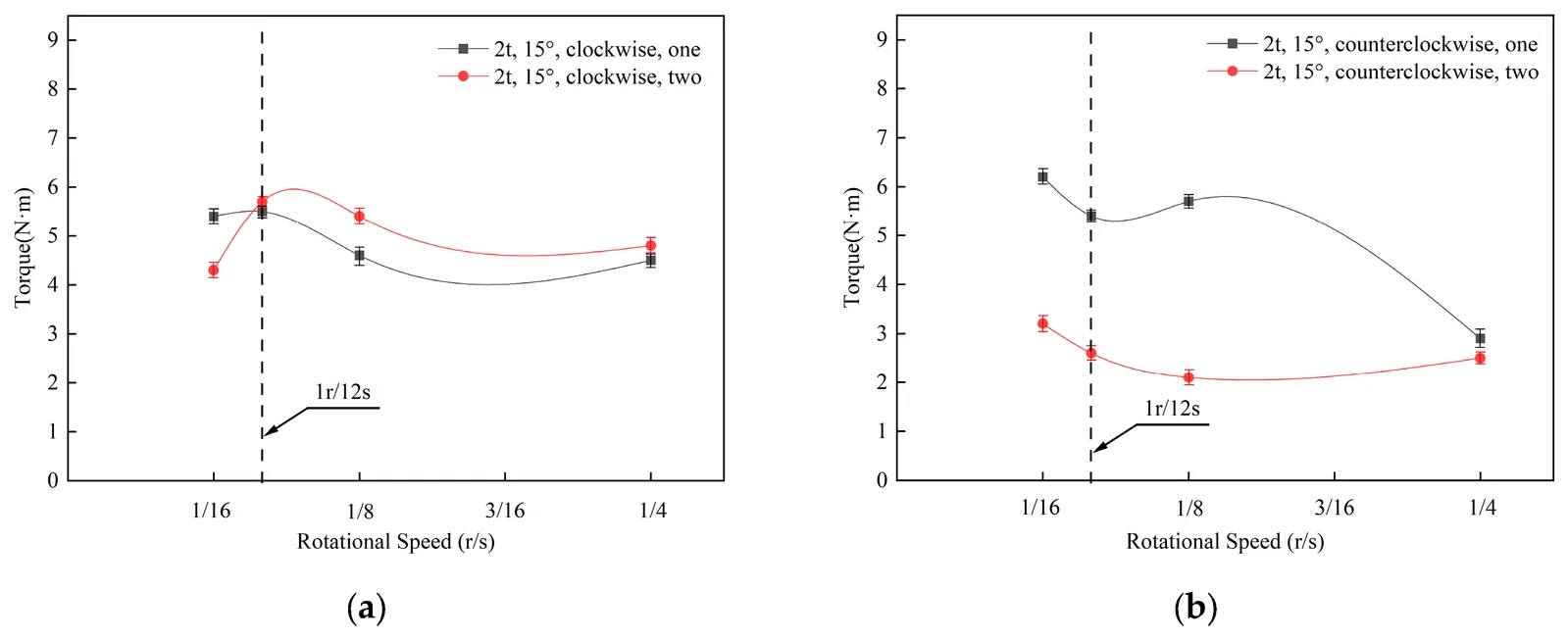
Torque variations in the single swivel eye and double series-connected swivel eye under the 2-ton load and the 15° offset angle: (**a**) clockwise and (**b**) counterclockwise.

**Figure 12:**
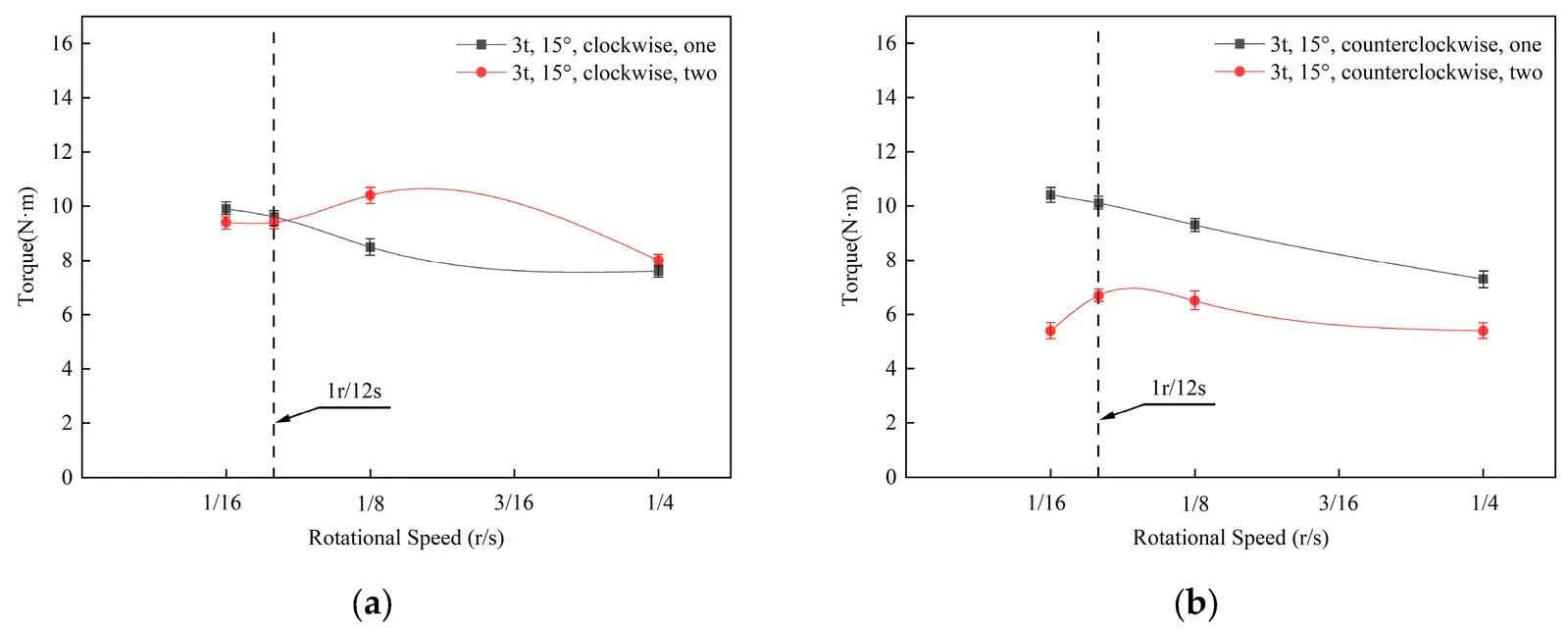
Torque variations in a single swivel eye and double series-connected swivel eye under the 3-ton load and the 15° offset angle: (**a**) clockwise and (**b**) counterclockwise.

Measurements using the double series-connected swivel eye were conducted under the following conditions: loads of 2 tons and 3 tons; rotational speeds of 1 rev/4 s, 1 rev/8 s, 1 rev/12 s, and 1 rev/16 s in both clockwise and counterclockwise directions; and an offset angle of 15°. A total of 16 test conditions were evaluated, and the results for the single swivel eye and the double series-connected swivel eye under the corresponding conditions were compared in Figure 11 and Figure 12, and listed in Table 6.

As shown in Figure 11 and Figure 12, under the 2-ton and 3-ton loads, respectively, during clockwise rotation, the torques borne by the single and double series-connected swivel eyes are similar, while during counterclockwise rotation, the double series-connected swivel eye shows better torque cancelation than the single swivel eye. Still, the maximum torque transmitted to the top of the double series-connected swivel eye is far below the specified limit of 100 N·m.

### 4.3. Discussion of Torque Measurement Characteristics

The above results show that the proposed test system can distinguish torque differences caused by load, rotational direction, rotational speed, offset angle, and connection configuration. The torque range remains small relative to the 100 N·m design requirement, but the changes among different operating conditions are still clear. This indicates that the sensing system is not only a pass/fail verification tool, but also a useful platform for identifying the torque envelope of the coupled connector assembly.

The difference between clockwise and counterclockwise rotation is mainly related to the combined influence of the rotation cancelation mechanism, contact friction at the pin-connected interfaces, and the load eccentricity introduced by the offset angle. When the load increases from 2 tons to 3 tons, the torque level also increases. This suggests that the measurement system can capture load-dependent torque behavior without changing the mechanical configuration of the test platform.

The comparison between the single swivel eye and the double series-connected swivel eye further reflects the role of connection configuration in torque transmission. The two-swivel configuration does not simply reduce torque under all conditions; instead, its effect depends on rotational direction and speed. This result is useful for connector design because it shows that the number and arrangement of swivel elements should be evaluated together with realistic rotation and offset-angle conditions.

The value of the proposed measurement method is that tensile force, torque, rotational speed, and offset angle are controlled or acquired within the same test system. This provides a repeatable way to evaluate coupled torque transmission in heavy-load rotary connectors and gives direct reference values for the torque design of shipborne helicopter vertical replenishment equipment.

## 5. Concluding Remarks

In summary, a real-time torque measurement test system was developed for coupled swivel-eye connections used in shipborne helicopter vertical replenishment. By using this system, the torque responses of single and double series-connected swivel eyes were measured under different loads, offset angles, rotational speeds, and rotation directions. The obtained results provide a basis for evaluating the torque transmission behavior and connection safety of swivel-eye assemblies under heavy-load rotary conditions. The system uses a load cell, torque transducer, and tachometer to measure load, torque, and rotational speed, and transmits the data via Wi-Fi to the integrated data processing device. The system covers multiple operating conditions for vertical replenishment connectors. Using this system, the torques of a single swivel eye and a double series-connected swivel eye after rotation decoupling were measured and compared. The results reveal the main torque patterns at the top of the swivel eye. Under all tested conditions, the maximum measured torque is no greater than 12 N·m, which is well below the 100 N·m limit. The results validate the torque-bearing performance of the swivel eyes used for shipborne helicopter vertical replenishment and provide experimental reference values for the torque design of external lifting equipment and its connection points with the helicopter.

## Figures and Tables

**Table 1:** Measurement range of the test system.

Torque (N·m)	Tensile Force (kN)	Rotation Speed (rev/min)	Offset Angle (°)
0~200	0~100	0~50	0~45

**Table 2:** Tension force and torque sensor measurement parameters.

Parameter	Range	Maximum	Resolution	Relative Error
Tension force	0~100 kN	150 kN	10 N	1%
Torque	0~200 N·m	300 N·m	0.03 N·m	1%

**Table 3:** DH5908L dynamic strain gauge technical specifications.

Channel Number	A/D Resolution	Sampling Rate	Communication	Communication Distance	Power Duration
4 × 16	24 bits	50 kHz	Wi-Fi	200 m	12 h

**Table 4:** Average torque under varied rotation speed for the single swivel eye (N·m).

Offset Angle (°)	2-Ton Load		3-Ton Load	
	Clockwise	Counterclockwise	Clockwise	Counterclockwise
0	5.450	2.475	8.875	5.125
5	6.075	2.625	10.125	4.900
10	5.425	2.450	9.325	4.575
15	5.000	5.050	8.900	9.275
Average	5.488	3.150	9.306	5.969

**Table 5:** Average torque under varied offset angle measured for the single swivel eye (N·m).

Rotation Speed (rev/s)	2-Ton Load		3-Ton Load	
	Clockwise	Counterclockwise	Clockwise	Counterclockwise
1/16	5.425	3.500	9.600	5.750
1/12	5.700	3.225	9.175	6.300
1/8	5.425	3.350	9.925	6.275
1/4	5.400	2.525	8.925	5.550
Average	5.488	3.150	9.306	5.969

**Table 6:** Average torque under varied load and rotation speed measured for both the single swivel eye and double series-connected swivel eye under the offset angle of 15° (N·m).

Rotation Speed (rev/s)	2-Ton Load		3-Ton Load	
	Single Swivel Eye	Double Series-Connected Swivel Eye	Single Swivel Eye	Double Series-Connected Swivel Eye
1/16	4.463	3.750	7.675	7.400
1/12	4.463	4.150	7.738	8.050
1/8	4.388	3.750	8.100	8.450
1/4	3.963	3.650	7.238	6.700
Average	4.319	3.825	7.688	7.650

## Data Availability

Data are available from the corresponding author upon reasonable request.
